# Optimizing Salt Leakage Mitigation and Comparing Sorption–Desorption Characteristics of Polyacrylamide-Based Hydrogels

**DOI:** 10.3390/polym16040525

**Published:** 2024-02-15

**Authors:** Yimo Liu, Zhongbao Liu, Zhipeng Qie, Zepeng Wang, Weiming Sun

**Affiliations:** Department of Environment and Life, Beijing University of Technology, Beijing 100124, China; nxlym@emails.bjut.edu.cn (Y.L.); qiezhipeng@bjut.edu.cn (Z.Q.); ln1wangzepeng@163.com (Z.W.); swimming5734@gmail.com (W.S.)

**Keywords:** atmospheric water harvesting, polyacrylamide, LiCl, water adsorption, salt leakage

## Abstract

Solid hygroscopic materials are extensively utilized in diverse fields, including adsorption heat transfer, adsorption heat storage, atmospheric water harvesting (AWH), and air conditioning dehumidification. The efficacy and energy efficiency of these materials in practical applications are significantly influenced by their adsorption and desorption properties. Yet, the introduction of inorganic salts to boost adsorption performance can result in issues like salt leakage. In this research, we prepared a polyacrylamide hydrogel through free radical polymerization, and its water-absorbing capabilities were improved by incorporating the hygroscopic salt lithium chloride. We compared it to a salt-based porous adsorbent, AlFum-LiCl, which also exhibited strong water adsorption properties and the potential for large-scale production. While AlFum-LiCl suffered from limited pores and salt leakage during high water uptake, the optimized PAM-LiCl displayed superior water sorption capabilities, showing no salt leakage even at water uptake of up to 3.5 g/g. At 25 °C, PAM-LiCl achieved equilibrium water uptake of 1.26 g/g at 30% RH and 3.15 g/g at 75% RH. In this context, utilizing 20 g of PAM-LiCl for the AWH experiment yielded daily water outputs of 8.34 L/kg at 30% RH and 16.86 L/kg at 75% RH. The salt-optimized PAM-LiCl hydrogel offers the benefit of application in higher relative humidity environments without the risk of deliquescence, underscoring its promise for atmospheric water harvesting.

## 1. Introduction

With the intensification of climate change and pollution of water supplies, many parts of the world face the problem of water shortage, which seriously threatens people’s livelihood [1,2]. By 2050, approximately 2 billion people will be living in areas of absolute water scarcity owing to limited freshwater resources and a surging demand for water [3]. Desalination and atmospheric water harvesting (AWH) technologies are being developed to address this challenge [4,5]. The atmosphere contains over 13,000 km^3^ of public renewable water resources [6]. AWH technology is not affected by geography and hydrology [7], so it is more flexible and diverse in solving water use problems. The corresponding methods include mist trapping [8], surface cooling [9], and sorption atmospheric water harvesting (SAWH) [10,11,12]. By contrast, SAWH is different from other water intake technologies owing to its minor environmental restrictions, a wide range of applicable humidity, and high efficiency because of the advancement of material science.

The performance of SAWH largely depends on the hygroscopic and desorption properties of the adsorbent. In various applications, silica gel, zeolite, and activated carbon [13,14,15] are commonly used porous materials. However, they generally suffer from low equilibrium adsorption capacity and high energy consumption for desorption. Metal–organic frameworks (MOFs) can have high porosity and good water absorption through rational composition and pore structure design [16,17,18,19,20]. However, the equilibrium hygroscopic amount and the range of adsorption humidity are still limited. Their adsorption performance is often improved by compounding with other hygroscopic salts, such as LiCl and CaCl_2_ [10,21,22,23,24]. The porous matrix can help the hygroscopic salts disperse in the pores and prevent deliquescence from affecting the adsorption and desorption kinetics [25]. However, the pores of porous materials are limited, and there is still a risk of salt leakage and corrosion when working under high relative humidity.

The presence of several hydrophilic groups in the hydrogel, such as -OH and -NH_2_ [26], can simultaneously reduce the evaporation enthalpy required for water evaporation [27]. However, the poor performance at low humidity makes it necessary to combine hygroscopic factors (high affinity for water vapor, including inorganic hygroscopic salts, organic hydrophilic polymers, and other active ions) in applications [28,29,30,31,32,33]. Incorporating hygroscopic salts into the hydrogel matrix is a promising way to improve the dynamic adsorption performance [34,35]. Of course, locking in the hygroscopic salts in more than low-humidity conditions in the spotlight is also a challenge [36].

Polyacrylamide (PAM) hydrogels are linear polymers obtained by polymerizing acrylamide (AM) monomers. PAM has stable physical/chemical properties and characteristics, such as low toxicity and environmental friendliness. PAM also exhibits excellent hydrophilic properties, owing to its intrinsic nitrogen/oxygen-contained functional groups with high polarity. Furthermore, PAM has hydrogen bonds formed between oxygen-contained groups and water molecules.

The literature has documented the incorporation of hygroscopic salts like LiCl and CaCl_2_ into PAM to enhance its hygroscopic properties in composites [37,38]. At equivalent temperatures and molar concentrations, LiCl solutions have lower surface water vapor partial pressures than CaCl_2_, indicating LiCl’s superior hygroscopicity [39]. In a 2022 study published in Advanced Materials, Yu et al. [38] thoroughly investigated the water sorption and desorption performance of PAM-LiCl at low relative humidity. When modulating the salt content, PAM-LiCl displayed no salt leakage with water uptake reaching 2.5 g/g. Additionally, water sorption reached 1.5 g/g at 25 °C and 30% RH. When applying 200 mg of the material at 25 °C and 20% RH, daily water extraction achieved 7.1 g/g, with an air water extraction rate of 0.51 g/g per cycle.

Based on the previous studies, we further explored the adsorption as well as salt leakage properties of PAM-LiCl, which were categorized into the following aspects:

1. Through the optimization of salt content, we identified component ratios that effectively function across a broad spectrum of relative humidity, minimizing salt leakage while maintaining robust adsorption–desorption performance. Concurrently, we analyzed and characterized the distinctive adsorption mechanism of superabsorbent hydrogels, which differs from that of porous adsorbents, and conducted tests to evaluate their adsorption–desorption efficacy.
2. We observed a disparity between the hydrogel’s theoretical water adsorption capacity (expressed in g/g) and its actual water absorption (measured in g). To address this, we devised an environmentally friendly method for synthesizing a substantial volume of PAM (approximately 500 mL, as demonstrated in this study). AWH experiments were conducted, loaded with 20 g of PAM-LiCl, under varying humidity conditions—specifically, low (30% RH) and high (75% RH) humidity. The optimal daily water yield achieved was 16.77 g/g of material, amounting to a total of 335 mL per day. This significantly exceeds the standard 1–2 g material mass typically reported in existing literature for AWH experiments utilizing hydrogels.

## 2. Materials and Method

### 2.1. Materials

The chemicals for PAM-LiCl, including AM, (Analytical Reagent, AR), were purchased from Shanghai Macklin Biochemical Co., Ltd., Shanghai, China. *N*,*N′*-Methylenebis (2-propenamide) (MBA, Chemically Pure, CP), *N*,*N*,*N′*,*N′*-tetramethylethylenediamine (TEMED, AR), potassium persulfate (KPS, AR), and lithium chloride (LiCl, AR) were purchased from Aladdin Industrial Company, Shanghai, China.

The reagents used for the chemical synthesis of AlFum were aluminium sulphate (Al_2_(SO_4_)_3_⋅18H_2_O, 99%), sodium hydroxide (NaOH, AR), and fumaric acid (CO_2_H–CH=CH–CO_2_H, 99%), which were purchased from Aladdin Industrial Company, Shanghai, China. All chemicals were used as purchased without further purification. 

### 2.2. Methods of Materials Preparation

Fabrication of PAM-LiCl gel: AM was added to deionized water and sonicated for 1 min to prepare a 10% AM solution. Moreover, nitrogen gas was purged for 10 min to thoroughly remove the dissolved oxygen in the water. Under sonication, 0.8 mL, 3.8 mg/mL MBA solution, 0.4 mL, 50 mg/mL KPS solution, and 100 µL TEMED were rapidly added to every 10 mL AM solution. After a 12 h period to allow the PAM to fully gel, it was washed with deionized water, pre-frozen for 12 h, and then freeze-dried for 24 h. The lyophilized PAM was soaked in 10, 20, 25, and 30 wt% LiCl solutions for 24 h and then dried at 80 °C for 72 h to obtain PAM-LiCl-10, PAM-LiCl-20, PAM-LiCl-25, and PAM-LiCl-30, respectively. In this study, we polymerized around 500 mL of AM solution in a single batch, producing 500 mL of watery PAM hydrogels. Using multiple containers allows for the production of greater quantities of PAM hydrogels from a singular batch.

Fabrication of AlFum-LiCl: Al_2_(SO_4_)_3_⋅18H_2_O (2.91 g) was mixed in DI water (10 mL) and stirred for 30 min under the condition of a constant temperature water bath at 70 °C until it was denoted as solution A. Fumaric acid (1.01 g) and NaOH (1.055 g) were dissolved in water (15 mL) and stirred for 15 min until complete dissolution. This solution was denoted as solution B. B was dropped into A and stirred for 3 h to obtain the AlFum solution. The above solution was allowed to stand, centrifuged, and washed with water three times. The crude product was collected after drying at 80 °C for 10 h and then impregnated with 10 and 20 wt% LiCl solutions to prepare AlFum-LiCl-10 and AlFum-LiCl-20, respectively. AlFum-LiCl powder was obtained by activation in a vacuum oven at 100 °C for 8 h [21].

### 2.3. Characterization

Scanning electron microscope (SEM) images were acquired using a Czech TESCAN MIRA LMS to observe the morphology and microstructure of the samples, including the energy dispersive spectrometer (EDS) energy spectrum. X-ray diffraction (XRD) patterns were acquired using an X-ray diffractometer (Bruker D8 Advance, Mannheim, Germany) at a scan rate of 10°/min. The functional group composition of the gel was examined by Fourier-transform infrared spectroscopy (FTIR, Thermo Scientific Nicolet iS20, Waltham, MA, USA). The salt content of the gel was determined using a thermogravimetric (TG) analyzer (Rigaku TG/DTA8122, Tokyo, Japan). The hygroscopic properties of the adsorbent were measured using a multi-station weight method of gas vapor sorption (BSD-DVS, Shenzhen, China). The Li^+^ concentration of the trapped water was tracked through inductively coupled plasma–mass spectrometry (IPC-OES MS, Agilent 7700X, Santa Clara, CA, USA). The porosity of AlFum-LiCl was measured using a physical adsorption analyzer (ASAP 2020HD88, Virginia Beach, VA, USA)

The dynamic adsorption characteristics of adsorbents are essential factors that determine the adsorption rate and affect the cycle time. Standard dynamic adsorption models mainly include the LDF model, which is widely used in the study of adsorption kinetics of adsorbents owing to its simple model form. The LDF model is a single-resistance model, which assumes that the adsorption rate of the adsorbent is proportional to the difference in concentration of the adsorbate outside the adsorbent particle. That is, the resistance of the external diffusion process can be neglected compared with the resistance of the internal diffusion, and the expression of the model is as follows:

(1) $$\frac{dx}{dt}=K\left(x_{eq}-x_{t}\right),$$

where *dx*/*dt* is the adsorption rate; *x_t_* is the dynamic adsorption capacity, g/g; and *x_eq_* is the equilibrium adsorption capacity, g/g. Integrating both sides of the above equation, we obtain the following:

(2) $$\frac{x_{t}-x_{0}}{x_{eq}-x_{0}}=1-e^{-Kt},$$

where *x*_0_ represents the initial adsorption amount, g/g. For adsorption processes, this value is generally zero because the test samples are always dried before adsorption testing. Rearranging Equation (2) and applying the Nabier logarithm, we obtain the following:

(3) $$-\ln\left(1-\frac{x_{t}}{x_{eq}}\right)=Kt.$$

The atmospheric water harvesting experiments were conducted in a constant temperature and humidity chamber. The chamber allowed adjustments of the dry bulb temperature (DB) and RH as needed, and uniform temperature and humidity were maintained by an airflow with a speed of 2.15 m/s. The mass change in the hydrogel and the amount of condensed water were measured using a precision electronic balance (ME802E) with a mass measurement error range of ±0.001 g. The amount of water uptake was calculated using Equation (4):

(4) $$W_{up}=\frac{m_{1}-m_{2}}{m_{2}}.$$

where $m_{1}$ represents the mass of the hydrogels after moisture absorption, and $m_{2}$ denotes initial mass before absorption.

## 3. Results and Discussion

### 3.1. Principle of Water Vapor Sorption on PAM-LiCl Hydrogel

PAM hydrogel, a linear polymer, is synthesized from acrylamide monomers through a polymerization process. Characterized by stable physicochemical properties, non-toxicity, and environmental compatibility, PAM hydrogel possesses numerous reactive functional groups. Its capacity for easy chemical modification and forming hydrogen bonds with water molecules endows it with superior hydrophilicity and water-retention capabilities. The preparation of PAM hydrogels is typically achieved through free radical polymerization. To ensure uniform distribution of LiCl within the gel matrix, a PAM-LiCl hydrogel composite adsorbent was synthesized. This involved freeze-drying the PAM hydrogel and subsequently integrating LiCl via the impregnation technique. It is noteworthy that the PAM-LiCl hydrogel is non-porous. Contrasting with solid adsorbents like silica gel, zeolite, and MOFs, which have limited pore space, polymer-based hydrogel adsorbents like PAM-LiCl exhibit the unique ability to swell considerably upon water absorption, storing the absorbed water within their expansive crosslinked polymer networks instead of in conventional pores. This characteristic enables the PAM-LiCl hydrogel to demonstrate enhanced equilibrium water vapor sorption capabilities. The water vapor sorption mechanism in the composite hygroscopic hydrogel encompasses both physical and chemical adsorption processes. Physical adsorption predominantly takes place between the water vapor and the porous matrix, where the vapor is captured within the porous structure via van der Waals forces. Nevertheless, this aspect of moisture adsorption contributes relatively minimally to the overall process. The primary mechanism in the hygroscopic process is the chemisorption of water vapor by LiCl, encompassing three stages: hydration reaction, liquid dissolution, and absorption. Initially, the crystalline LiCl within the hydrogel reacts with water vapor in a hydration reaction, resulting in the formation of a hydrated salt. Subsequently, these hydrated salts continue to absorb atmospheric moisture, which liquefies, forming a film of saturated salt solution on the surface of the hydrated salt particles. With ongoing water absorption, the hydrated salts dissolve entirely, culminating in the formation of a saturated salt solution. This solution persists in absorbing water vapor, leading to a gradual reduction in salt concentration and eventually resulting in a low-concentration salt solution. Throughout this process, the hydrophilic polymer chains in the PAM interact with the liquid water resulting from the deliquescence of LiCl, retaining this water within the swollen hydrogel matrix in forms of bound, weakly bound, and free water. Owing to its pronounced hydrophilicity and high water content, the polymer matrix of the PAM hydrogel not only promotes rapid water absorption and swelling but also effectively retains the absorbed water, preventing leakage. The synergistic effect of physical adsorption and chemical absorption mechanisms renders the composite hygroscopic hydrogel highly efficient in water vapor sorption, making it suitable for various applications demanding effective moisture control.

**Figure 1:**
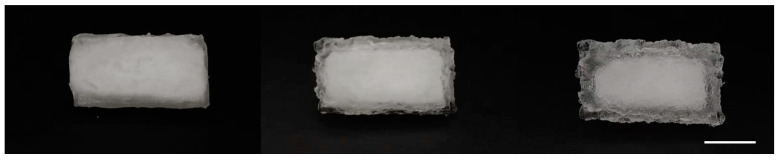
Photographs of PAM-LiCl before water capture (**left**), water uptake of 0.5 g/g (**middle**) and 1 g/g (**right**); Scale bar: 1 cm.

The observable changes in the bulk PAM-LiCl hydrogel before and after water uptake, as depicted in Figure 1, demonstrate its distinctive physical properties. Before absorbing moisture, the PAM-LiCl hydrogel is white and nearly opaque in appearance. This characteristic is due to its dense polymer structure in the dry state. Upon reaching a water uptake of 0.5 g/g and 1.0 g/g, respectively, the regions within 2 mm and 3.5 mm from the hydrogel edges were observed to become increasingly transparent. This shift in transparency is attributed to the integration of water molecules into the polymer backbone of the PAM-LiCl hydrogel, leading to alterations in its internal structure. As water molecules are incorporated into the polymer network and hydrogen bonds form, both the volume and transparency of the hydrogel increase. The observed expansion in volume and change in transparency serve as evidence that the PAM-LiCl hydrogel is capable of effectively adsorbing and storing water molecules. Practically, this characteristic renders the PAM-LiCl hydrogel a highly effective hygroscopic material, making it particularly well-suited for applications that demand high water absorption, such as in dehumidification systems and water treatment processes.

### 3.2. Materials Testing and Characterization

#### 3.2.1. Surface Morphology and Elemental Distribution

**Figure 2:**
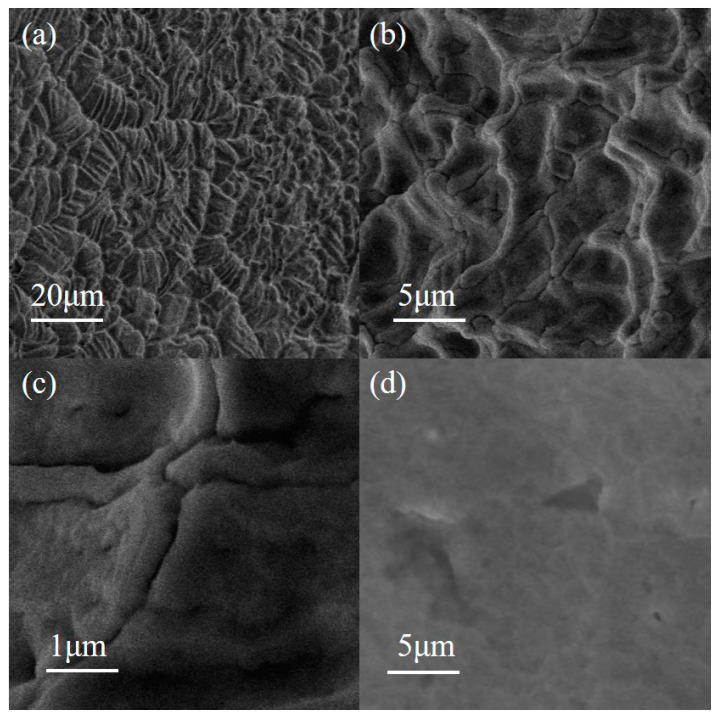
SEM images of PAM-LiCl before adsorption with scale bar of (**a**) 20 µm, (**b**) 5 µm, (**c**) 1 µm, and (**d**) after adsorption with a scale bar of 5 µm.

**Figure 3:**
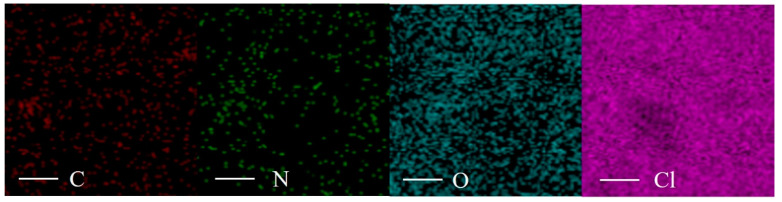
EDS images of PAM-LiCl.

SEM and EDS images of PAM-LiCl hydrogels were obtained using a TESCAN MIRA LMS scanning electron microscope from the Czech Republic, as depicted in Figure 2 and Figure 3, respectively. In its unabsorbed state, the surface of the dried PAM-LiCl hydrogel exhibited noticeable wrinkling (as illustrated in Figure 2a–c), likely a result of natural shrinkage and drying of the polymer network during its preparation. Conversely, the water-absorbed samples displayed a smoother surface in the SEM images (refer to Figure 2d). This alteration in surface texture is attributed to the swelling of the gel as it adsorbs water molecules, leading to the expansion of the polymer network and smoothing out the initial wrinkles. Furthermore, EDS analysis elucidated the distribution of elements such as C (carbon), N (nitrogen), O (oxygen), and Cl (chlorine) within the PAM-LiCl samples. The EDS analysis results indicated a uniform distribution of these elements within the PAM-LiCl matrix, corroborating the homogeneous incorporation of LiCl within the PAM matrix, as demonstrated in Figure 3.

#### 3.2.2. Chemical Functional Group

**Figure 4:**
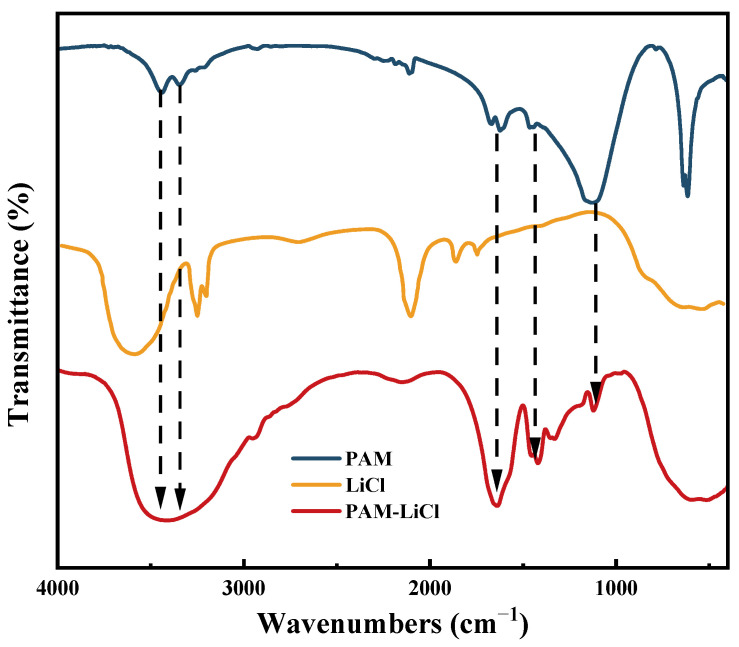
FTIR spectra of PAM, LiCl and PAM-LiCl.

FTIR was employed to demonstrate the chemical properties of PAM and the PAM-LiCl hydrogel, and Figure 4 shows the results. The feature at 3439.09 cm^−1^ is attributed to the vibration of free -NH_2_, and that at 3345.97 cm^−1^ is attributed to conjugation -NH_2_. The signal at 1668.21 cm^−1^ can be assigned to the C=O stretching vibration of amide I, corresponding to the carbonyl group, and the characteristic at 1622.93 cm^−1^ is attributed to N-H bending vibration, corresponding to amide II; the signal at 1121.74 cm^−1^ can be attributed to the in-plane wobble vibration of -NH_2_ in the amide moiety. A search for standard FTIR profiles reveals that the above characteristics of the PAM hydrogels prepared through chemical cross-linking generally agree with the standard PAM profiles. The red curve represents the IR spectrum of PAM-LiCl, in which the characteristic peaks of PAM and LiCl can be found, further proving the success of the PAM-LiCl composite.

#### 3.2.3. Crystal Structure

**Figure 5:**
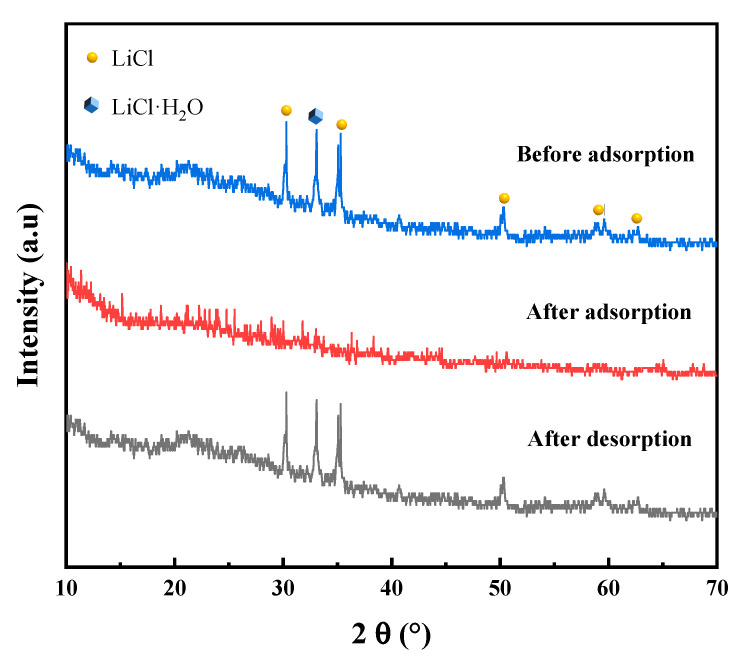
XRD diffraction spectra of PAM-LiCl before and after water uptake.

XRD analysis is a pivotal technique for examining material structures, elucidating both crystalline and amorphous properties. Figure 5 illustrates the XRD results of PAM-LiCl hydrogel, both before and after adsorption. In the XRD pattern, PAM-LiCl displays a broader band at approximately 2θ 20°, indicative of its amorphous character. Such an amorphous structure, commonly observed in polymer-based materials, suggests the absence of long-range atomic ordering in the material. The XRD pattern prior to adsorption reveals sharp peaks, corresponding to crystalline LiCl and LiCl∙H_2_O. This finding indicates the presence of LiCl in its crystalline form within the unabsorbed PAM-LiCl. Upon water absorption by the PAM-LiCl hydrogel, the crystalline phase peaks vanished, suggesting complete dissolution of LiCl crystals and the existence of Li and Cl ions within the PAM backbone. This transition exemplifies the deliquescence of LiCl during water absorption, highlighting its ability to capture atmospheric water vapor and convert it into liquid water. Following the desorption process and water removal, LiCl recrystallizes, leading to the reappearance of sharp peaks in the XRD patterns. This outcome denotes a dynamic change in the crystalline state of LiCl throughout the adsorption–desorption cycle of PAM-LiCl. Collectively, these findings underscore the critical role of LiCl in the adsorption behavior of PAM-LiCl hydrogels. The crystallization dissolution recrystallization cycle of LiCl significantly contributes to the volume expansion and water absorption characteristics of PAM-LiCl hydrogels. The interaction of liquid water with the hydrophilic polymer chains in the PAM, facilitated by hydrogen bonding, leads to efficient water storage.

### 3.3. Optimization of Hygroscopic Salt Content

As highlighted in Section 3.1’s analysis of the adsorption principle of the PAM-LiCl hydrogel, the hygroscopic salt LiCl is pivotal to the hydrogel’s adsorption performance. In practice, however, the concentration of the hygroscopic salt influences not only the adsorption performance but also the potential for salt leakage from the adsorbent. This dual effect arises because, although a higher salt content can enhance the adsorbent’s equilibrium water uptake in low relative humidity settings, it concurrently heightens the risk of salt leakage in high-humidity conditions. This risk is predominantly attributed to the finite water storage capacity of the PAM polymer backbone. Consequently, optimizing the salt content in PAM-LiCl hydrogels is essential to adapt them for a broader spectrum of environmental conditions. By achieving an equilibrium between the hygroscopic salt content and the water storage capacity of the PAM polymer backbone, one can enhance the adsorbent’s performance across various humidity conditions and mitigate the risk of salt leakage. This optimization process will entail integrating the hygroscopic properties of LiCl with the physical constraints of the PAM matrix to ascertain the optimal salt concentration, thereby enabling the adsorbent to demonstrate efficient adsorption capacity and stability under diverse environmental conditions.

**Figure 6:**
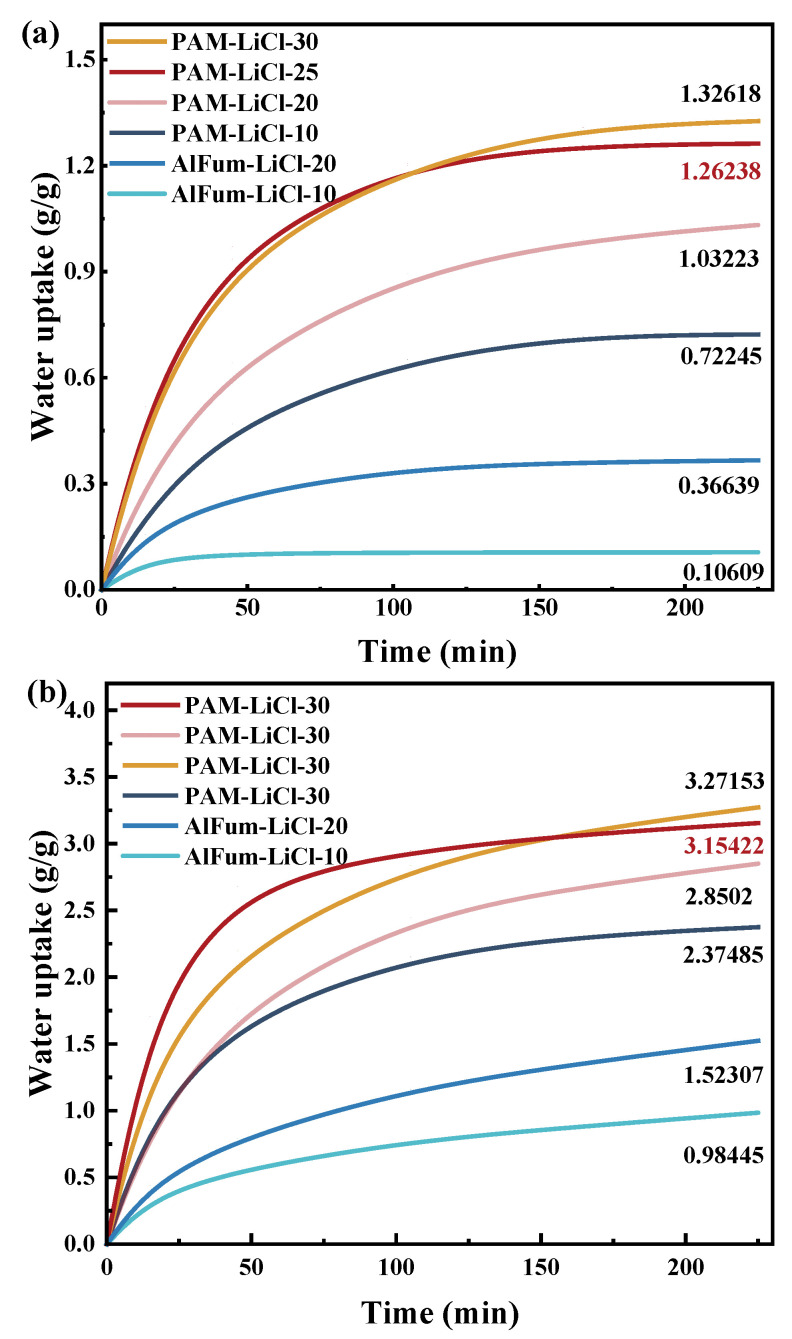
The adsorption performance of PAM-LiCl and AlFum-LiCl with different salt contents at 25 °C, 30% RH (**a**) and 75% RH (**b**).

Material characterization results in Section 3.2 confirmed the successful incorporation of the hygroscopic salt LiCl into the PAM hydrogel, laying the groundwork for further optimization of the material’s salt content. PAM-LiCl with different salt contents were prepared and named PAM-LiCl-10, PAM-LiCl-20, PAM-LiCl-25, and PAM-LiCl-30, according to the concentration of the impregnated LiCl solution (10–30 wt%). Except for PAM-LiCl, another commonly employed adsorbent, aluminium fumarate, was selected to compare the adsorption, desorption, and salt leakage performance. We prepared AlFum and compounded AlFum with 10 and 20 wt% LiCl as AlFum-LiCl-10 and AlFum-LiCl-20, respectively. Finally, we discussed the results together. The two materials were crushed into a powder of approximately 50 µm and passed through a 200 mesh screen using a grinder before the materials were tested. Adsorption tests were carried out on the samples at 25 °C, low humidity (30% RH) and high humidity (75% RH), as shown in Figure 6a,b.

**Figure 7:**
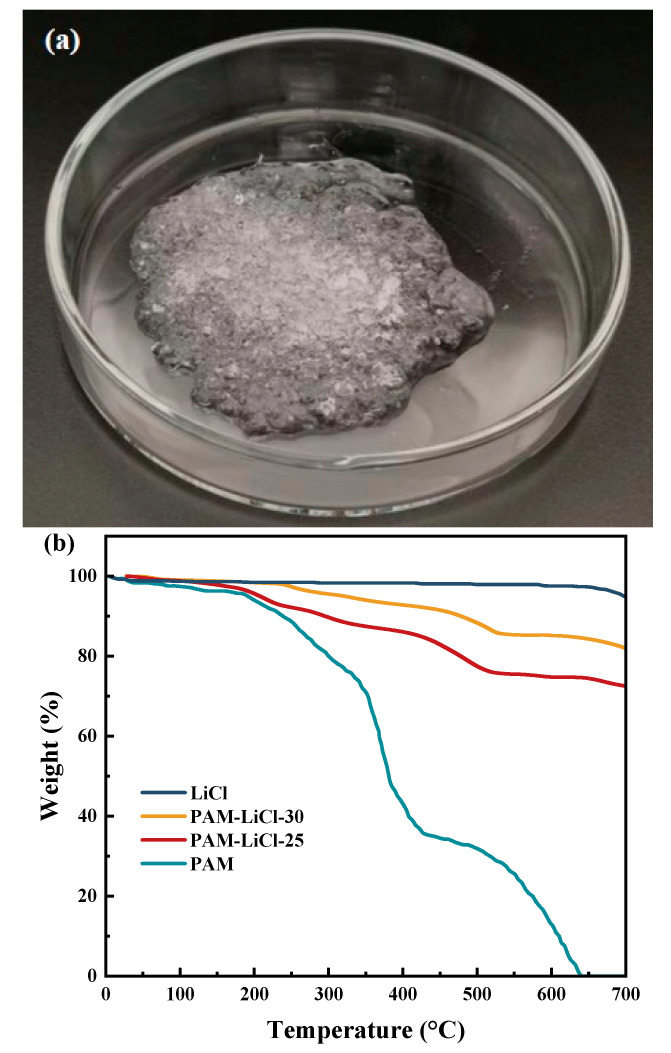
(**a**) Salt leakage trend occurring when the water uptake of PAM-LiCl reaches 3.27 g/g (**b**) TG analysis of PAM, PAM-LiCl-25, PAM-LiCl-30, and LiCl.

Regardless of the low- or high-humidity conditions, when the concentration of impregnated LiCl increased (10–25 wt%) owing to the rise in LiCl content, the hygroscopicity, equilibrium adsorption capacity, and average adsorption rate at equilibrium (225 min) of PAM-LiCl gradually increased. The equilibrium moisture uptake of PAM-LiCl-25 reached 1.26 and 3.15 g/g at 25 °C, 30% and 75% RH, respectively, with average adsorption rates of 5.6 and 14 mg/g·min. Under the same conditions, the equilibrium water uptake of PAM-LiCl-10 was 0.72 and 2.37 g/g, respectively, with average adsorption rates of 3.2 and 10.5 mg/g·min. When the LiCl concentration was further increased to 30 wt%, although the equilibrium adsorption capacity was higher than that of PAM-LiCl-25, the average adsorption rate decreased to a certain extent. At 25 °C and 75% RH for 45 min, the average adsorption rate of PAM-LiCl-25 was 59.3 mg/g·min, whereas that of PAM-LiCl-30 was 48.6 mg/g·min. The possible reason for this phenomenon is that too much LiCl induces aggregation and prevents exposure of the adsorption site, resulting in a reduced contact area with air, which is consistent with the findings of Lu et al. [38]. In addition, PAM-LiCl-30 reaches hygroscopic equilibrium at 75% RH, that is, a risk trend of salt leakage when the adsorption reaches 3.27 g/g (Figure 7a). After treatment by LiCl solution with various concentrations, the actual mass of the LiCl substance loaded on PAM was further investigated. To investigate the above reasons, the LiCl content in the PAM-LiCl-25 and PAM-LiCl-30 was obtained through TG analysis, as shown in Figure 7b. The LiCl content of PAM-LiCl-25 was 72 wt%, whereas that of PAM-LiCl-30 was 82 wt%. The reason is that the polymer hydrogel stores the captured liquid water in its swollen cross-linked polymerization network when absorbing moisture. Thus, the hydrogel acts as a reservoir during the moisture absorption process. When the salt content is relatively high, the hydrogel percentage becomes less, and the reservoir naturally holds less water. When moisture absorption reaches the solid water storage limit of the PAM-LiCl hydrogel, it will liquefy, similar to the porous–salt composite adsorbent saturated with moisture. However, the amount of water uptake required for salt leakage to occur will be much more significant for the hydrogels. From another aspect, for operation at higher moisture contents, the salt content only needs to be further optimized to ensure the moisture absorption capacity of the PAM-LiCl hydrogel whilst also taking care of the water storage capacity of the PAM, thereby limiting the leakage of hydrated salt and helping to avoid potential corrosion in practical applications.

**Figure 8:**
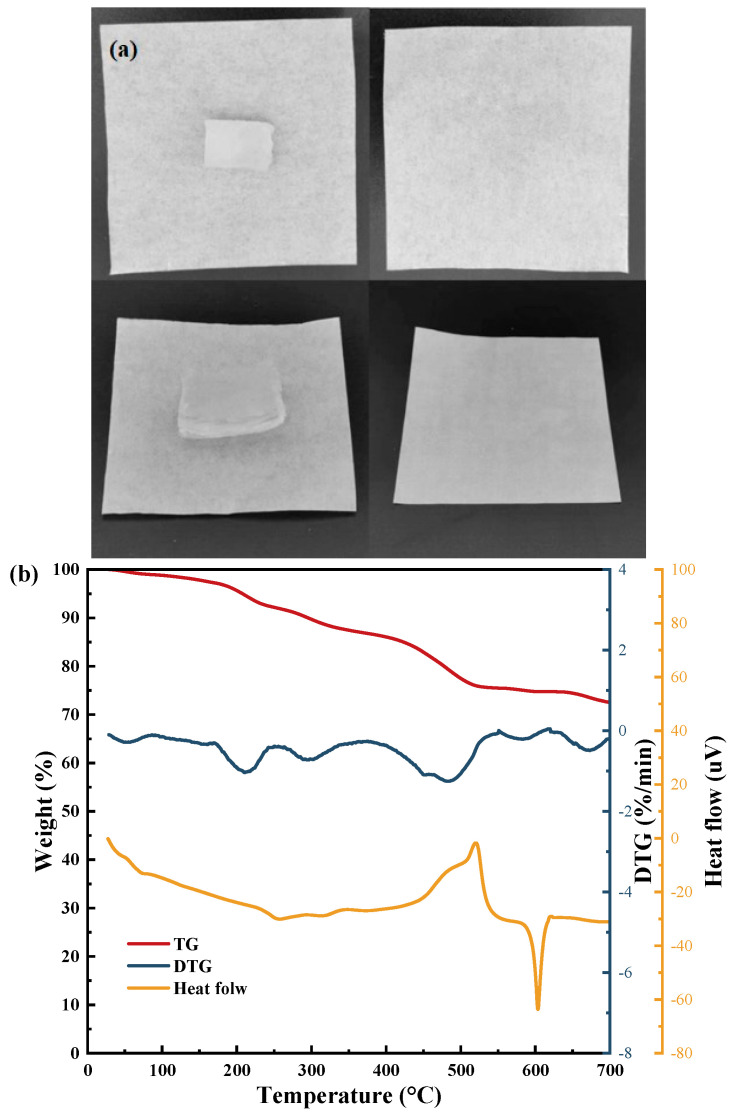
(**a**) Photos of PAM-LiCl without salt leakage at 1.5 g/g (**top**) and 3.5 g/g (**bottom**) of water uptake (**b**) TG, DTG, and heat flow curves of PAM-LiCl-25.

Combining these two reasons with the objective climatic conditions for air extraction applications in most parts of the world, PAM-LiCl-25 was selected as the optimal sample for subsequent experiments. Moreover, salt leakage was not observed at water uptake up to 3.5 g/g, as shown in Figure 8a. Figure 8b depicts the TG, DTG, and heat flux of PAM-LiCl-25.

**Figure 9:**
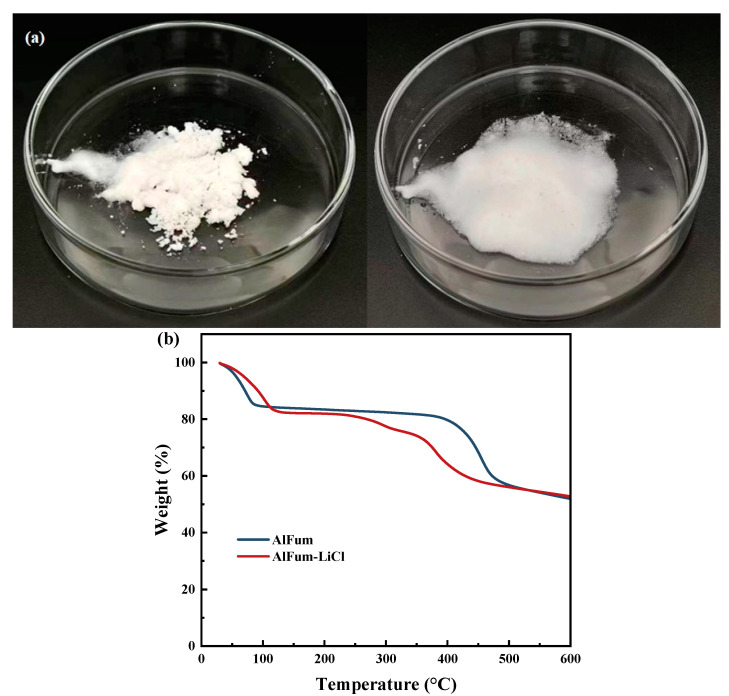
(**a**) Salt leakage of AlFum-LiCl at 1.0 g/g (**left**) and 1.5 g/g (**right**) of water uptake (**b**) TG analysis of AlFum and AlFum-LiCl.

The findings from the aforementioned study on AlFum-LiCl have also indicated that increased salt content does not invariably lead to improved outcomes. AlFum-LiCl-20, prepared through impregnation with 20 wt% concentration of LiCl, already showed liquid dissolution when water uptake reached 1.0 g/g (Figure 9a). Increasing the mass fraction of LiCl to increase the equilibrium moisture uptake is not advisable. Therefore, AlFum-LiCl-20 was selected as the target sample for subsequent discussion. Figure 9b shows the TG analysis of AlFum and AlFum-LiCl. Concurrently, given that AlFum-LiCl is a porous MOF-salt-based adsorbent, it facilitates both physical and chemical adsorption during the moisture adsorption process. Consequently, we assessed the pore sizes of AlFum and AlFum-LiCl using a physical adsorption analyzer, and have presented the findings in Table 1 [21].

### 3.4. Absorption and Desorption Performance Test and Comparison

#### 3.4.1. Adsorption Isotherm

**Figure 10:**
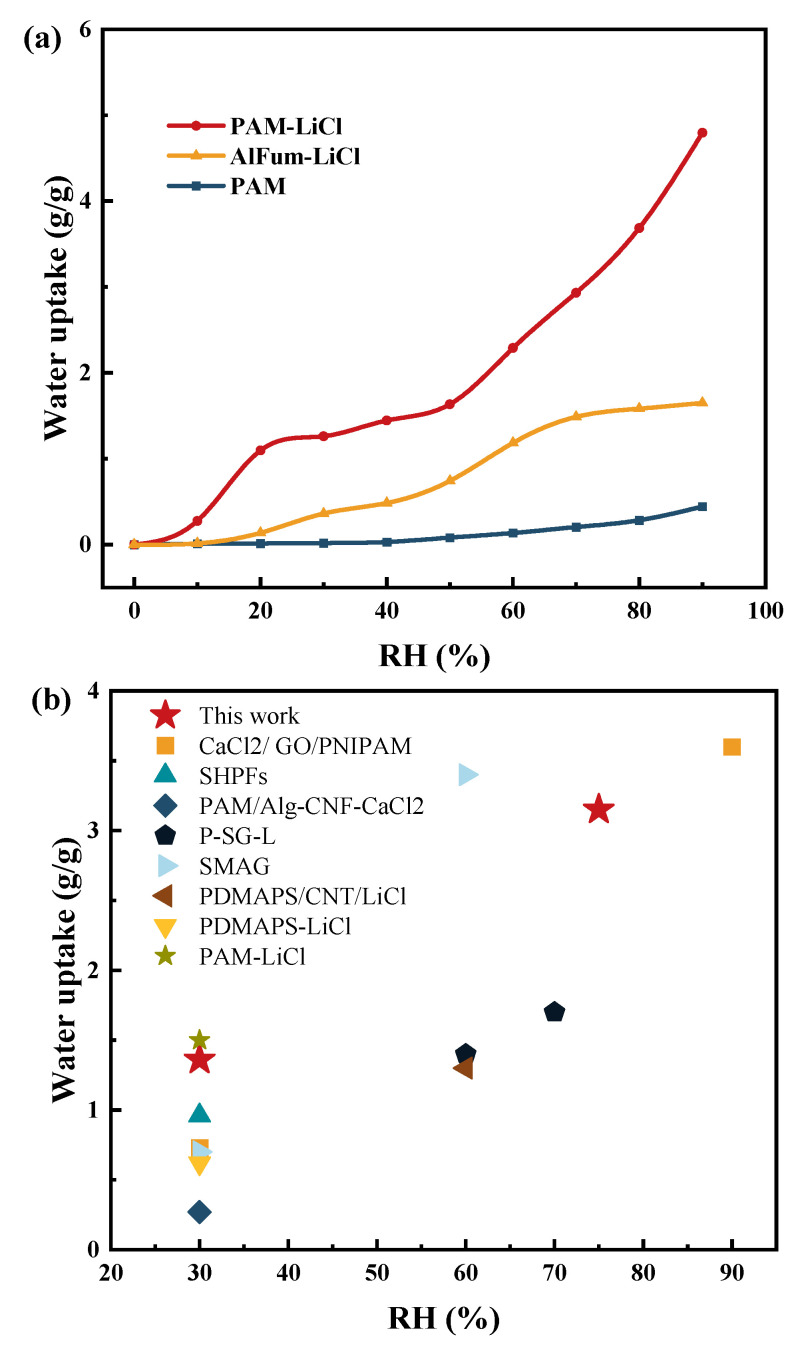
(**a**) Adsorption isotherms of PAM, AlFum-LiCl, and PAM-LiCl at 25 °C (**b**) The water uptake of advanced hydrogel adsorbents reported in recent years was compared with that of PAM-LiCl.

Figure 10a shows the adsorption isotherms for PAM, PAM-LiCl, and AlFum-LiCl at 25 °C. Pure PAM has almost no sorption capacity in the low humidity range because of the lack of sorption sites, with a more significant enhancement in the high humidity range, but only up to 0.44 g/g (RH = 90%). The adsorption process of AlFum-LiCl on water vapor can be divided into two stages: when 10% < RH < 70%, the adsorption of AlFum-LiCl on water vapor increases continuously; when 70% ≤ RH ≤ 90%, the adsorption capacity of AlFum-LiCl on water vapor gradually tends to saturate with little change. The underlying mechanism involves AlFum-LiCl hygroscopic salts undergoing both physisorption and chemisorption. This process entails physisorption at matrix surface sites, formation of salt hydrates, deliquescence of these hydrates, and vapor absorption by the resulting aqueous salt solutions. At low relative humidity, water molecules are chemisorbed in a single molecular layer on the surface of LiCl; as the relative humidity increases, water molecules will be physisorbed on the non-salt surface, that is, the AlFum pores. Moreover, together with the chemisorption occurring on the surface of LiCl, a multi-molecular layer is formed, blocking the humid air. Another important reason is that the pores of solid adsorbents, such as AlFum, are limited, and their adsorption capacity reaches a bottleneck with increasing RH. PAM-LiCl compensates for both of these disadvantages in terms of moisture absorption: it has moisture absorption active sites and good water storage capacity. In the low RH range, the hygroscopic properties of PAM-LiCl are satisfactory. From 10% RH, the hygroscopic ability increases rapidly from 0.27 to 1.09 g/g at 20% RH as LiCl starts to deliquesce. From 30% to 90% RH, the equilibrium hygroscopic capacity of PAM-LiCl increases from 1.26 g/g to a dramatic value of 4.79 g/g. Moreover, the water uptake of PAM-LiCl increases monotonically with RH increases owing to the water storage and swelling capacity of PAM. The hygroscopic performance is significantly higher than AlFum-LiCl, including PAM in static terms. Meanwhile, Figure 10b shows the adsorption performance of advanced adsorbents reported in recent years along with PAM-LiCl. For more specific information, see Table 2. The hygroscopic attributes of PAM-LiCl notably surpass those of reported porous materials under both low- and high-humidity conditions. Typically, the water uptake of porous MOFs and salt-based MOFs was under 1 g/g at 25 °C and 30% RH. While the PAM-LiCl in this work performed well among super-adsorbent hydrogels, its water uptake is marginally lower than Yu et al.’s findings (1.36 < 1.47 g/g, 25 °C, 30% RH) because of its different salt content. However, the optimized salt concentration ensures that the PAM-LiCl presented here can function at elevated RH levels without salt leakage concerns, which is an additional advantage.

#### 3.4.2. Dynamic Adsorption Test

**Figure 11:**
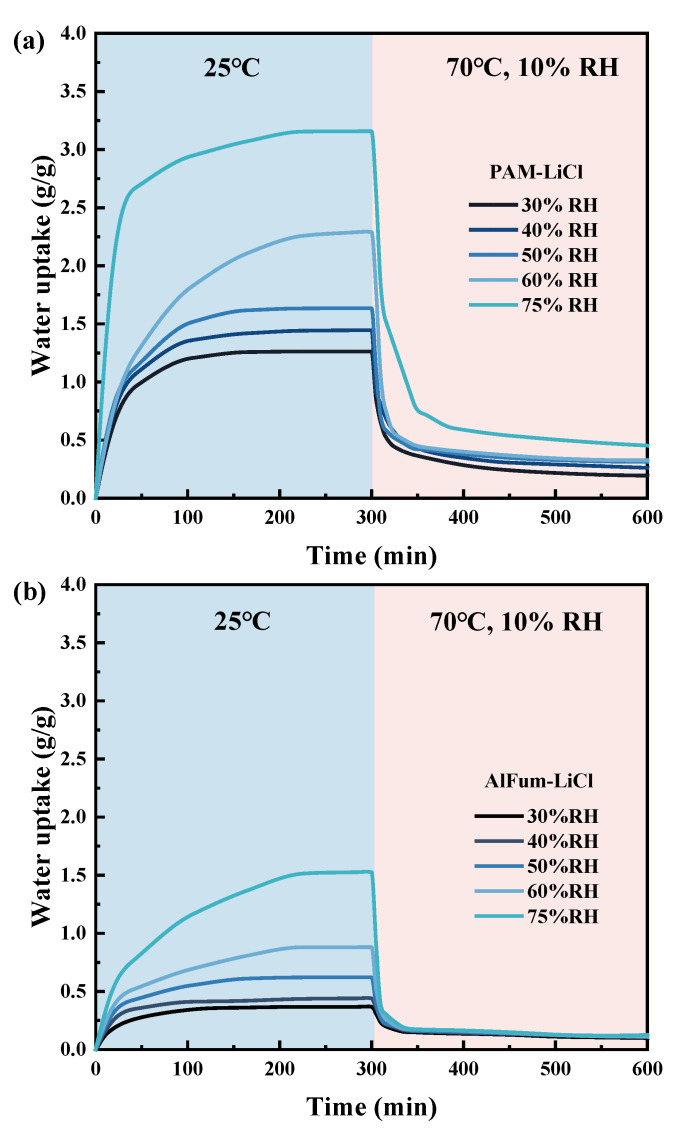
Dynamic adsorption–desorption curves of PAM-LiCl (**a**) and AlFum-LiCl (**b**) at 25 °C, 30%, 40%, 50%, 60%, and 75% RH adsorption, and 70 °C, 10% RH desorption.

Static adsorption curves alone do not indicate the dominance of PAM-LiCl in AWH applications; the adsorption kinetics is equally critical. Figure 11a illustrates the curves of PAM-LiCl adsorption at 25 °C with different relative humidities and subsequent desorption at 70 °C with 10% RH (water vapor partial pressure approximately equal to the saturation vapor pressure at 25 °C). At various relative humidities, 80% more saturated adsorption capacity could be achieved after 100 min adsorption. With an increase in relative humidity, the amount of water collected after adsorption equilibrium increases from 1.26 g/g at 30% RH to 3.15 g/g at 75% RH. Moreover, the corresponding average adsorption rate also increased from 4.43 to 11.05 mg/g·min. From another aspect, the desorption kinetics are satisfactory, and PAM-LiCl can desorb 80% or more of the adsorbed water within 60 min. Comparatively, the adsorption amount and the adsorption kinetics of AlFum-LiCl are slower (Figure 11b), particularly at higher relative humidity. At 25 °C, 75% RH, the equilibrium water uptake of AlFum-LiCl was 1.52 g/g, and the average adsorption rate was 5.35 mg/g min.

**Figure 12:**
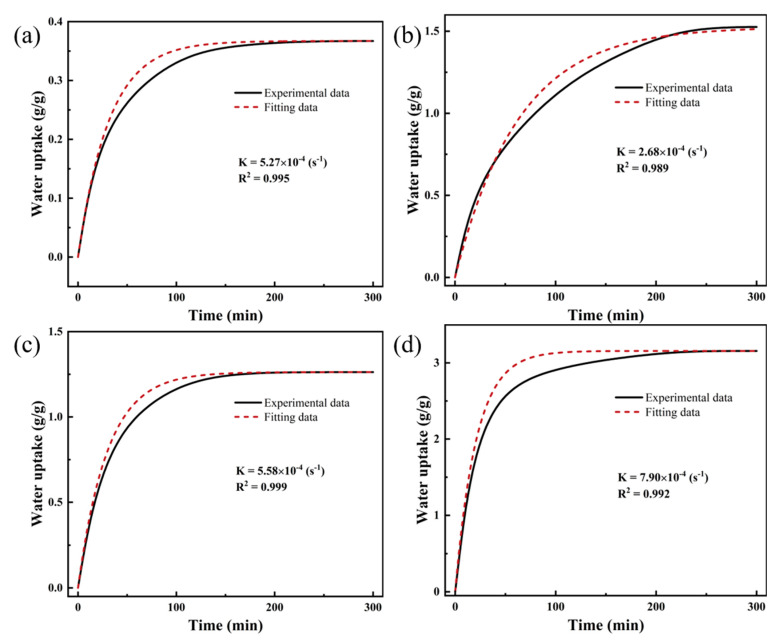
Comparison of experimental data of AlFum-LiCl and PAM-LiCl with LDF fitting (**a**) AlFum-LiCl (25 °C, 30% RH) (**b**) AlFum-LiCl (25 °C, 75% RH) (**c**) PAM-LiCl (25 °C, 30% RH) (**d**) PAM-LiCl (25 °C, 75% RH).

We also fit the experimental data of PAM-LiCl and AlFum-LiCl with the linear driving force (LDF) model to investigate their adsorption rate coefficients to understand further the dynamic adsorption characteristics of these two adsorbents. The adsorption rate coefficients K of PAM-LiCl at 25 °C 30% RH and 75% RH are, respectively, 5.58 × 10^−4^ (s^−1^) and 7.90 × 10^−4^ (s^−1^), both of which are better than AlFum-LiCl. Furthermore, the R^2^ achieved through LDF fitting consistently surpasses 0.99, providing evidence of well-fitting results. Detailed information can be found in Figure 12 and Table 3. The results indicate that PAM-LiCl not only exhibits outstanding equilibrium water uptake but also demonstrates enhanced adsorption kinetics when compared to the salt-based porous material, AlFum-LiCl. When the moisture absorption of AlFum-LiCl reaches a certain level, the subsequent water vapor adsorption process inevitably becomes slower by the influence of the deliquescent water layer on the surface. Thus, AlFum-LiCl reaches the adsorption equilibrium more rapidly at low relative humidity, that is, the adsorbent surface does not undergo deliquescence when the equilibrium adsorption is low. Correspondingly, the PAM-LiCl skeleton prevents the formation of a dense crystalline layer on the surface of the hygroscopic material during desorption. Thus, the water molecules stored inside the gel are transported to the material surface uniformly and orderly.

#### 3.4.3. Dynamic Desorption Test

**Figure 13:**
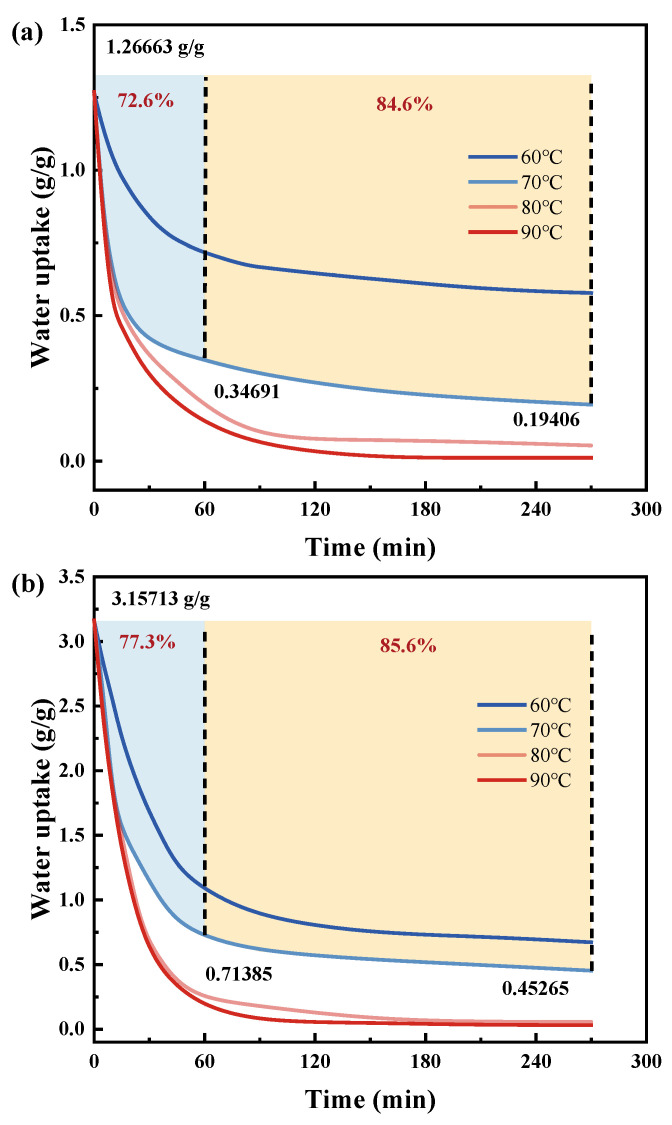
Desorption curves at 60 °C, 70 °C, 80 °C and 90 °C for PAM-LiCl at a water uptake of about (**a**) 1.26 g/g (i.e., saturated at 25 °C, 30% RH adsorption) (**b**) 3.15 g/g (i.e., saturated at 25 °C, 75% RH adsorption).

Figure 13 shows the desorption data of PAM-LiCl at different temperatures after reaching an adsorption equilibrium at 30% and 75% RH, respectively. Undoubtedly, the higher the temperature, the faster the desorption rate. After reaching the equilibrium adsorption of 1.26 g/g at 25 °C and 30% RH, PAM-LiCl can desorb 72.6% of the adsorbed water in 60 min and 84.6% of the captured water in 270 min at a reasonable desorption temperature of 70 °C. Of course, if the desorption temperature is appropriately increased to 80 °C or 90 °C, the desorption perfection will be higher. Notably, the desorption rate of PAM-LiCl does not slow down and even increases somewhat at 25 °C, 75% RH with an equilibrium adsorption capacity as high as 3.15 g/g. At a desorption temperature of 70 °C, the desorption perfection of PAM-LiCl reached 77.3% after 60 min and 85.6% after 270 min, both of which were higher than that of the low water content case. The reason is that PAM-LiCl hydrogels possess the property of water absorption volume expansion. Moreover, the volume and specific surface area of PAM-LiCl increase with water absorption before reaching the water storage limit for liquefaction and salt leakage, leading to faster desorption kinetics. From another aspect, although the interaction between PAM-LiCl and trapped water makes more water molecules in the form of weak or free water [38], bounded water is also present. This case increases free water in the gel skeleton when a large amount of water is adsorbed compared with the PAM-LiCl gel, which adsorbs a small amount of water. The enhancement is less pronounced with the increase in desorption perfection, the specific surface area of PAM-LiCl, and the decrease in the proportion of weakly bound water.

Another reason for choosing 70 °C for regeneration is that although 80 °C, 90 °C has a higher perfection of desorption, with 94.6% perfection of regeneration at 60 min of desorption at 90 °C. At this time, a small percentage of the bound water in the gel skeleton is also desorbed. In practice, retaining a small percentage of bound water in the gel skeleton during desorption may be a more energy-efficient strategy so that the water absorbed and desorbed by PAM-LiCl is weakly bound. The desorption data demonstrate the good ability of PAM-LiCl to transport the stored water to the surface of the hydrogel. Moreover, there is no need to worry about the desorption and recirculation ability of PAM-LiCl after the adsorption of large amounts of water.

#### 3.4.4. Cyclic Adsorption and Desorption Test

**Figure 14:**
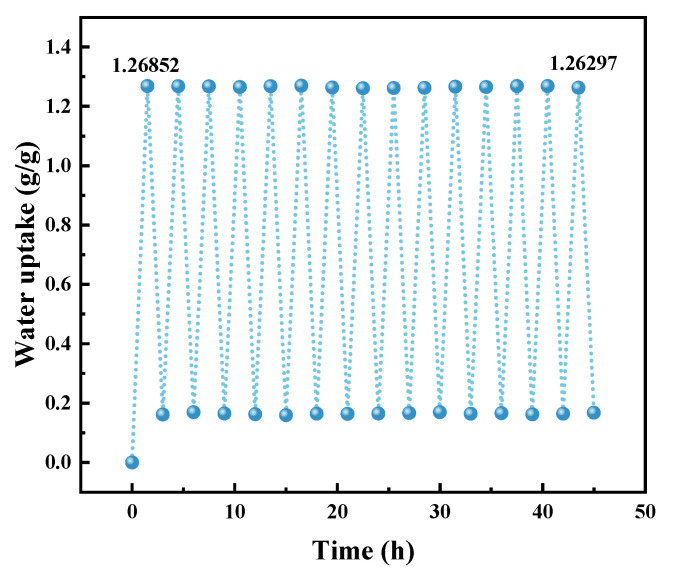
The cycling stability experiment of PAM-LiCl.

To assess the cyclic stability of PAM-LiCl, it underwent testing with a multi-station gravimetric gas vapor sorbent system (DVS). This involved adsorption at 25 °C and 30% RH for 90 min, followed by desorption at 70 °C and 10% RH for 90 min. Conducted over 15 consecutive cycles, the test revealed no significant degradation in the adsorption–desorption performance of PAM-LiCl, with results presented in Figure 14. Notably, the water uptake in the final cycle was only marginally lower, by 0.006 g/g, compared to the initial cycle. This finding indicates robust cyclic stability of the material, demonstrating its ability to maintain adsorption–desorption efficiency throughout multiple cycles.

### 3.5. Laboratory Atmospheric Water Harvesting Experiment

**Figure 15:**
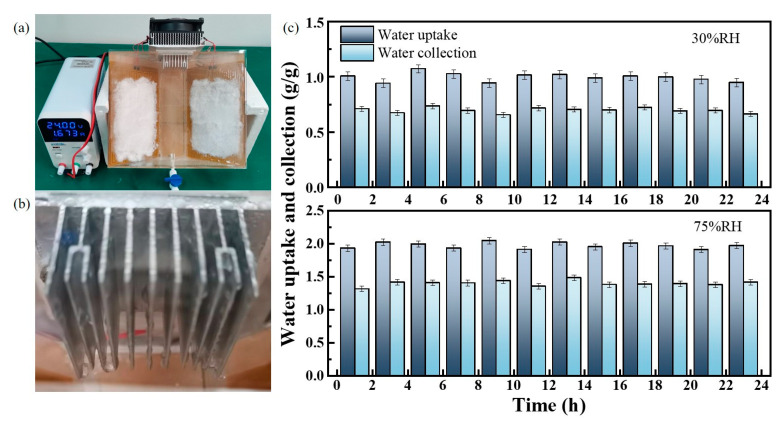
AWH performance of PAM-LiCl. (**a**) Photograph of the AWH device loaded with 20 g PAM-LiCl. (**b**) Photo of condensed clean water droplets on the condenser. (**c**) Water uptake and water harvesting at 25 °C, 30% RH (**top**) and 75% RH (**bottom**). Error bar: Standard deviation, SD.

Figure 15a presents a small intermittent AWH experiment bench built for this study, which mainly consists of a chamber composed of an acrylic box (the top cover can be opened manually during water sorption process), a DC power supply, a semiconductor cooling sheet, and a polyimide heating sheet. The DC power supply can precisely control the heating temperature based on the feedback from the thermocouple attached to the back of the heating plate; after heating, the water vapor condenses into liquid water at the fins of the semiconductor cooling sheet (see Figure 15b), and rolls down to the reservoir. A total of 20 g of activated PAM-LiCl was used in the experiment and placed on two 180 × 120 mm heating sheets. Owing to the viscosity of the gel material after a small amount of water uptake, PAM-LiCl fit well and tightly with the heating sheets. Then, in a constant temperature and humidity chamber, the air–water extraction experiments at 25 °C, 30% RH and 25 °C, 75% RH, were carried out. During the investigation, adsorption was carried out for 1 h, followed by desorption at a temperature of 70 °C for 1 h. Figure 15c shows the experimental results. After PAM-LiCl captures moisture at 30% RH, the average water collection is 0.69 g/g. After capturing moisture at 75% RH, the average water collection is 1.40 g/g. The small intermittent water intake cycle can perform 12 AWH cycles a day, and the daily water production reaches 8.34 l/kg at 30% RH and 16.86 l/kg at 75% RH owing to the fast adsorption and desorption kinetics of PAM-LiCl. The calculated average water collection efficiency (ratio of water collection to water absorption) is approximately 69%. In addition, we tested the Li^+^ content of the water collected by PAM-LiCl, and the average was approximately 0.34 ± 0.03 ppm (mg/L). When normalized to the daily water intake, the Li^+^ content intake (0.68–1.02 mg) was less than the recommended value (3.1 mg) [40]. From another aspect, although a slightly larger scale application of AWH experiments inevitably weakens the heat and mass transfer performance of PAM-LiCl, making its moisture uptake and water extraction less desirable than in the material experiments, the intermittent water extraction device applying 20 g of the material still has a competitive water production capacity (167 mL/day at 25 °C, 30% RH and 337 mL/day at 25 °C, 75% RH), which is sufficient to demonstrate its feasibility for larger scale applications. In addition, the potential of PAM-LiCl for wide-area ambient humidity applications, the economical nature of the materials required for synthesis, and the simplicity and scalability of synthesis make it a promising candidate for future sustainable implementation. However, after absorbing moisture, PAM exhibits increased viscosity, posing challenges in its granulation for use in multi-bed adsorption systems. Moreover, when PAM-LiCl powder is employed on a large scale, its volume expansion and heightened viscosity post-hydration often lead to material agglomeration. This agglomeration impedes the mass transfer channels, thereby substantially affecting the adsorption–desorption efficiency. Consequently, identifying a suitable carrier for PAM-LiCl becomes a critical consideration for its practical deployment on a larger scale.

## 4. Conclusions

In conclusion, by modulating the salt content, we prepared the hydrogel PAM-LiCl that demonstrated no salt leakage even with water uptakes of up to 3.5 g/g. Additionally, PAM-LiCl showcases efficient equilibrium water uptake and robust dynamic absorption/desorption capabilities, suitable for atmospheric water harvesting across diverse humidity levels. When contrasted with the salt-based porous adsorbent Alfum-LiCl, PAM-LiCl’s salt leakage threshold is 3.3 times superior. Water uptakes of 1.26 g/g at 25 °C, 30% RH, and 3.15 g/g at 75% RH by PAM-LiCl are, respectively, 3.5 and 2 times greater than those exhibited by Alfum-LiCl. At 75% RH, PAM-LiCl’s adsorption rate coefficient K stands at 7.90 × 10^−4^ (s^−1^), in contrast to Alfum-LiCl’s 2.68 × 10^−4^ (s^−1^). Coupled with its good desorption attributes, these findings underscore PAM-LiCl’s superiority for air water extraction over the comparative porous material, AlFum-LiCl. In AWH tests using 20 g of PAM-LiCl, our custom intermittent AHW device achieved daily outputs of 167 mL/day at 30% RH and 337 mL/day at 75% RH. This hints at its potential for scaled-up applications in atmospheric water harvesting.

## Figures and Tables

**Table 1:** Pore analysis of AlFum and AlFum-LiCl.

	S_BET_ (m^2^/g)	S_micro_ (m^2^/g)	S_micro_/ S_BET_	V_total_ (cm^3^/g)	D_ave_ (nm)
AlFum	506.4	415.1	82.0%	0.32	2.55
AlFum-LiCl	286.6	199.8	69.7%	0.19	2.65

**Table 2:** Summary of water sorption performance of reported sorbents of various types.

Type	Ref.	Author	Materials	Temperature (°C)	RH (%)	Water Uptake (g/g)
	This work		PAM-LiCl	25	30	1.36
					75	3.15
MOF	[11]	Tao Y 2023	TMM-Alfum	25	35	0.32
	[17]	Luo F 2023	MIL-160(Al) & MOF-303	25	30	0.44
					60	0.46
					90	0.56
Salt-based MOF	[10]	An H 2023	MOF-808-CACl_2_	25	30	0.56
	[21]	Liu ZB 2021	CM/GFP	27	40	0.54
					80	1.75
	[23]	Elsayed E 2019	MIL-101(Cr)/CaCl_2_	25	30	0.65
	[24]	Sun YY 2020	UiO-66-LiCl	25	30	0.6
					90	2.15
Hydrogels	[29]	Wang X 2022	CaCl_2_/GO/PNIPAM	25	30	0.73
					90	3.6
	[30]	Guo YH 2022	SHPFs	25	30	0.96
	[31]	Park H 2022	PAM/Alg-CNF-CaCl2	25	30	0.27
	[32]	Ma QL 2022	P-SG-L	20	60	1.4
					70	1.7
	[33]	Zhao F 2019	SMAG	25	30	0.7
					60	3.4
					90	6.7
	[35]	Aleid S 2022	PDMAPS/CNT/LiCl	25	60	1.3
					80	1.75
	[37]	Lei CX 2022	PDMAPS-LiCl	25	30	0.62
	[38]	Yu GH 2022	PAM-LiCl	25	20	1.47
					30	1.05

**Table 3:** Rate coefficients K and the square of correlation coefficients R2 for AlFum-LiCl and PAM-LiCl.

	Parameters	25 °C, 30%RH		25 °C, 75%RH	
Sample		K (s^−1^)	R^2^	K (s^−1^)	R^2^
Alfum-LiCl		5.27 × 10^−4^	0.995	2.68 × 10^−4^	0.989
PAM-LiCl		5.58 × 10^−4^	0.999	7.90 × 10^−4^	0.992

## Data Availability

Data available on request due to restrictions e.g., privacy or ethical. The data presented in this study are available on request from the corresponding author.
